# Developmental Time Course of SNAP-25 Isoforms Regulate Hippocampal Long-Term Synaptic Plasticity and Hippocampus-Dependent Learning

**DOI:** 10.3390/ijms21041448

**Published:** 2020-02-20

**Authors:** Katisha R. Gopaul, Muhammad Irfan, Omid Miry, Linnea R. Vose, Alexander Moghadam, Galadu Subah, Tomas Hökfelt, Christina Bark, Patric K. Stanton

**Affiliations:** 1 Department of Cell Biology & Anatomy, New York Medical College, Valhalla, NY 10595, USA; krgopaul@gmail.com (K.R.G.); irfanjadoon@icloud.com (M.I.); omidm7@stanford.edu (O.M.); linnea316@gmail.com (L.R.V.); alexander_moghadam@nymc.edu (A.M.); galadu_subah@nymc.edu (G.S.); 2 Department of Neuroscience, Karolinska Institutet, 171 77 Stockholm, Sweden; Toaffmas.Hokfelt@ki.se

**Keywords:** SNARE proteins, long-term potentiation, long-term depression, learning and memory, cognition, Schaffer collateral-CA1 synapses

## Abstract

SNAP-25 is essential to activity-dependent vesicle fusion and neurotransmitter release in the nervous system. During early development and adulthood, SNAP-25 appears to have differential influences on short- and long-term synaptic plasticity. The involvement of SNAP-25 in these processes may be different at hippocampal and neocortical synapses because of the presence of two different splice variants, which are developmentally regulated. We show here that the isoform SNAP-25a, which is expressed first developmentally in rodent brain, contributes to developmental regulation of the expression of both long-term depression (LTD) and long-term potentiation (LTP) at Schaffer collateral-CA1 synapses in the hippocampus. In one month old mice lacking the developmentally later expressed isoform SNAP-25b, Schaffer collateral-CA1 synapses showed faster release kinetics, decreased LTP and enhanced LTD. By four months of age, SNAP-25b-deficient mice appeared to have compensated for the lack of the adult SNAP-25b isoform, now exhibiting larger LTP and no differences in LTD compared to wild type mice. Interestingly, learning a hippocampus-dependent task reversed the reductions in LTP, but not LTD, seen at one month of age. In four month old adult mice, learning prevented the compensatory up-regulation of LTD that we observed prior to training. These findings support the hypothesis that SNAP-25b promotes stronger LTP and weakens LTD at Schaffer collateral-CA1 synapses in young mice, and suggest that compensatory mechanisms can reverse alterations in synaptic plasticity associated with a lack of SNAP-25b, once mice reach adulthood.

## 1. Introduction

Synaptosomal Associated Protein of 25kDa (SNAP-25) is one of three core SNARE (Soluble N-ethylmaleimide sensitive factor Attachment protein REceptor) proteins that participate in the fusion of the neurotransmitter-containing vesicles with the plasma membrane [1]. These three SNARE proteins interact to form a trimeric core-complex, and in the process overcome the energy barrier for plasma membrane fusion [2]. Protein–protein interactions between the SNARE fusion proteins, and with additional ancillary proteins, can modify the properties and alter vesicle fusion [3,4]. The other core SNARE proteins, synaptobrevin 2 or VAMP2 and syntaxin 1A, are anchored to the membrane via their C-terminal domains. In contrast, SNAP-25 tethers to the membrane via palmitoylation, which is a reversible and dynamic process [5,6,7], and participates in the formation of coiled–coil quaternary SNARE complexes, where alpha helices of all three proteins wrap around each other. In the nervous system, SNAP-25 plays a major role in neurotransmitter release [8], and during development, SNAP-25 has been suggested to play a role in promoting neurite outgrowth [9,10].

Alternative splicing is a mechanism that increases the diversity profile of protein coding genes. More than 95% of multiexon genes in higher eukaryotes go through alternative splicing [11,12,13]. SNAP-25 utilizes this process to produce two variants, SNAP-25a, which is the predominant isoform in neurons in mice during embryonic and early postnatal (PN) development, and SNAP-25b, which becomes the dominant isoform as central synapses mature into adulthood. During mRNA formation, the splicing machinery splices in either exon 5a or exon 5b, corresponding to SNAP-25a or SNAP-25b proteins, respectively. While the differences that affect SNAP-25a and SNAP-25b functionality are still being elucidated, there are clear phenotypic differences in mice expressing only SNAP-25a versus predominantly SNAP-25b [14,15]. The two splice variants differ by only nine out of 206 amino acids [16,17,18,19]. However, SNAP-25a is the major isoform peripherally in endocrine and neuroendocrine excitable cells throughout life and certain hypothalamic structures, but this still needs further thorough investigation [18,20,21]. Moreover, the differences between SNAP-25a and SNAP-25b span regions of the N-terminal amphipathic helix and the cysteine-rich linker region, spacing the two (N- and C-terminal) helices [19,22]. Likely, the different SNAP-25 isoforms change the tertiary structure of the core SNARE complex and thereby modify the affinities of SNARE-binding partner proteins. Additionally, SNAP-25 isoforms differentially interact with accessory proteins such as Munc18-1 and Gβγ subtypes [23].

Within the first week of life, in the mouse hippocampal formation, SNAP-25a is the dominant isoform that participates in vesicle fusion and development [18,24]. As mice mature, SNAP-25b and overall SNAP-25 levels increase dramatically in the entire brain. By three weeks of age, the level of SNAP-25b is more than 50% greater than SNAP-25a in the hippocampal formation. Additionally, between weeks 3 and 8, likely the time of synaptic maturation, there is a dramatic increase in the total amount of SNAP-25.

During the first two weeks of development, when SNAP-25a is the predominant isoform in brain, rodents exhibit predominantly, or entirely, metabotropic glutamate receptor (mGluR)-dependent forms of long-term depression (LTD) of synaptic transmission, while long-term potentiation (LTP) has yet to appear [25,26,27,28]. The timing of this change in relative levels of SNAP-25a to SNAP-25b mirrors the developmental upregulation of N-methyl-d-aspartate receptors (NMDARs), suggesting that SNAP-25a may play a greater role in the expression of mGluR-dependent LTD, while SNAP-25b may be more important for expression of NMDAR-dependent LTD and LTP. It is possible, but still undetermined, whether the switch in expression from SNAP-25a to SNAP-25b confers this ability to express LTD and LTP in older animals.

We have previously reported that, at four weeks of age, SNAP-25b-deficient mice demonstrate significantly reduced LTP and less ability to discriminate between intensities of presynaptic stimuli [29]. To evaluate the role of SNAP-25 isoforms on later developmental stages of synaptic transmission and plasticity, we studied this same strain of SNAP-25b-deficient mice, using one month and four month old animals, to compare and assess the early developmental and later adult impact of the absence of SNAP-25b. Normally, the switch in SNAP-25 mRNA isoforms happens during postnatal weeks 1–3 in the hippocampal formation, so in one month old wild type (WT) mice, a complete switch of the SNAP-25 proteins has occurred. We compared the magnitude of hippocampal LTP and LTD in in vitro hippocampal slices, and learning acquisition and memory retention in vivo, at these different ages, to assess the early developmental and long-term adult impact of the absence of SNAP-25b on synaptic plasticity and cognitive function.

## 2. Results

### 2.1. Vesicular Release Probability is Increased in One Month Old SNAP-25b-Deficient Mice

SNAP-25a and SNAP-25b are functionally different in their ability to facilitate exocytosis following association with the other core SNARE proteins in the SNARE complex [30]. Therefore, we analyzed whether SNAP-25a-only expressing mice exhibited functional differences in vesicular transmitter release properties by direct imaging of the release kinetics of the styryl dye FM1-43 using two-photon laser scanning microscopy. We compared SNAP-25b-deficient mice to wild type (WT) controls and assessed the effects of the lack of SNAP-25b on release kinetics at glutamatergic Schaffer collateral-CA1 terminals in hippocampal slices.

**Figure 1:**
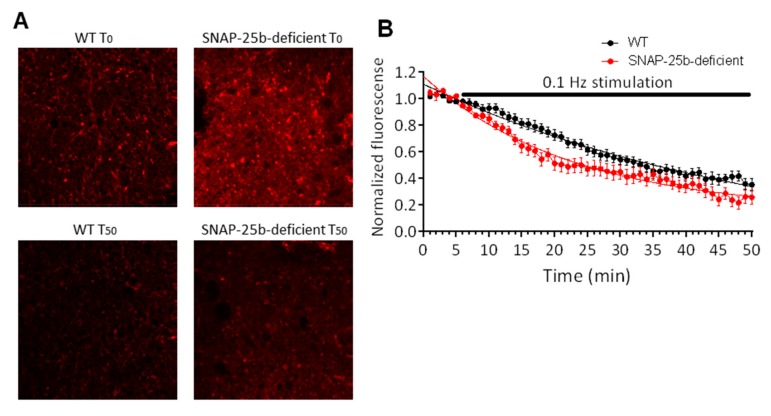
Presynaptic vesicular release from Schaffer collateral terminals in one month old SNAP-25b-deficient mice. (**A**) Representative fluorescent images of hippocampal field CA1 stratum radiatum in slices from WT and SNAP-25b-deficient mice prior to synaptic stimulation (T_0_) and after 50 min of 2Hz stimulation (T_50_). Bright puncta are presynaptic Schaffer collateral terminals. (**B**) Fluorescence decay of vesicular dye FM1-43 from Schaffer collateral-CA1 presynaptic boutons elicited by 0.1 Hz stimulation (black bar). SNAP-25b-deficient mice (filled circles, *n* = 20 puncta from 4 slices) showed a significantly faster rate of FM1-43 decay compared to wild type mice (open circles, *n* = 18 puncta from 6 slices). Each point is mean ± SEM of ‘n’ puncta.

To directly assess neurotransmitter release probability, Schaffer collateral presynaptic terminal vesicles were loaded with FM1-43, and the time course of fluorescent destaining was monitored in response to stimulus-evoked release of the dye using two-photon laser scanning microscopy. Schaffer collateral axons were stimulated with 0.1 Hz frequency to evoke neurotransmitter release. Schaffer collateral terminals in field CA1 of hippocampal slices from SNAP-25b-deficient mice showed significantly faster neurotransmitter release kinetics compared to the WT controls (Figure 1A), as measured by a faster rate of fluorescence decay of FM1-43. Fitting of destaining curves (Figure 1B) with a single exponential decay function Y = (Y0-NS)*exp(-K*X)+NS (Y = total vesicle brightness, Y0 = brightness at time 0, X = time, NS = brightness after end of stimulation period, K = rate constant in inverse units of X, half-life = 0.69/K) revealed significantly smaller half-life (13.7) and smaller decay time constant tau (0.05) values for SNAP-25b-deficient mice compared to WT half-life (23.3) and tau (0.031). Comparison of the two fits with extra sum-of-squares *F* test for the dissociation time constants (K) showed significant difference between SNAP-25b-deficient and wild type slices (*p* < 0.01).

### 2.2. Long-Term Potentiation and Long-Term Depression of Synaptic Strength

**Figure 2:**
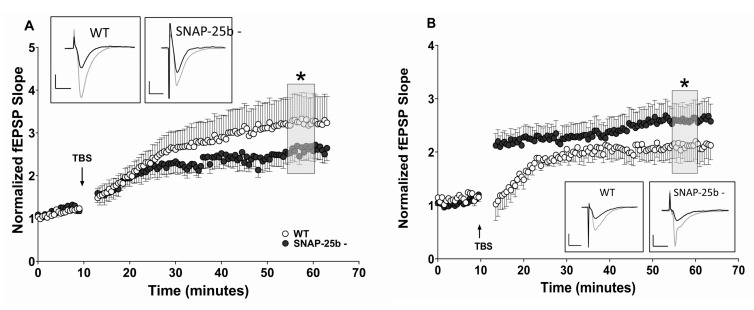
Alterations in stimulus-evoked LTP in SNAP-25b-deficient mice of both sexes at one and four months of age. (**A**) Theta burst stimulation (TBS)-induced LTP (burst data points not shown) at one month in hippocampal Schaffer collateral synapses. Arrows indicate application of TBS in each panel. Young SNAP-25b deficient mice expressed lower sLTP (filled circles, *n* = 13) compared to age-matched controls (open circles, *n* = 12). (* *p* < 0.01; Student’s *t*-test). Each point is normalized to the averaged baseline and is mean ± SEM of n slices. (**B**) Time course of LTP induced by TBS in littermate controls (open circles, *n* = 9) and SNAP-25b-deficient mice (filled circles, *n* = 9) in slices from 4 month old mice. Older SNAP-25b-deficient mice show enhanced LTP with an altered PTP compared to littermate controls (* *p* < 0.01; Student’s *t*-test). Each point is normalized to the averaged baseline and is mean ± SEM of n slices. LTP was calculated as mean of grey box, compared to pre-TBS normalized baseline. Insets: Representative Schaffer collateral-evoked fEPSPs in WT and SNAP-25b deficient slices immediately before (black traces) and 50 min after (grey traces TBS stimulation, with calibration bars in (**A**) 1mV/10msec, in (**B**) 2mV/10msec.

A high-frequency theta-burst stimulus (TBS) was used to induce stimulus-dependent LTP (sLTP) at Schaffer collateral-CA1 synapses in the hippocampal slices from one and four month old mice. At one month of age, sLTP was reduced in SNAP-25b-deficient mice compared to age-matched WT littermate controls (Figure 2A). The reduced expression of LTP suggests an important role for the missing SNAP-25b isoform in the expression of LTP. However, there clearly is LTP retained that does not require presence of SNAP-25b.

In contrast to slices from one month old mice, at four months of age SNAP-25b-deficient mice displayed LTP that was significantly larger than LTP in WT control mice (Figure 2B). In addition to expressing augmented LTP, four month old SNAP-25b deficient mice exhibited larger rapid post-tetanic potentiation of fEPSPs (PTP) shortly after induction of high-frequency stimulation (HFS), in contrast to the reduced PTP in one month old mice. While one month old mice lacking SNAP-25b displayed reduced LTP, the older animals, exhibited enhanced LTP compared to age-matched controls. This is clear evidence for presynaptic compensatory mechanisms that upregulate LTP in absence of the switch from SNAP-25a to SNAP-25b.

**Figure 3:**
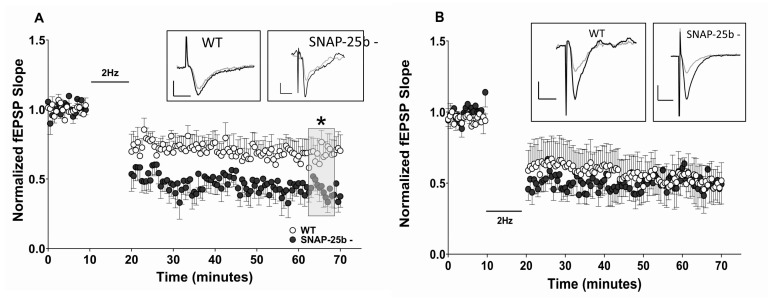
Changes in LTD at Schaffer collateral-CA1 synapses in hippocampal slices from one and four month old SNAP-25b-deficient mice of both sexes. (**A**) LTD at one month of age at Schaffer collateral-CA1 synapses induced by a 2Hz LFS. SNAP-25b-deficient mice (filled circles, *n* = 8) exhibited significantly greater LTD compared to controls (open circles, *n* = 7). Bar indicates application of LTD stimulus (2Hz/10min, data points not shown). (* *p* < 0.01; Student’s t-test). Each point is normalized to the averaged baseline and is mean ± SEM of n slices. (**B**) After prolonged absence of SNAP-25b, four month old SNAP-25b-deficient mice (filled circles, *n* = 8) displayed LTD similar to WT littermates (open circles, *n* = 8). Each point is normalized to the averaged baseline and is mean ± SEM of n slices. LTD was calculated as mean of grey box, compared to pre-TBS normalized baseline. Insets: Representative Schaffer collateral-evoked fEPSPs in WT and SNAP-25b deficient slices immediately before (black traces) and 50 min after (grey traces) LBS stimulation, with all calibration bars 1mV/10msec.

Due to the essential roles posited for both LTP and LTD in learning and memory, we also examined stimulus-evoked LTD of synaptic transmission (sLTD) in mice lacking SNAP-25b at one and four months of age. At one month, but not four months, of age, hippocampal slices from SNAP-25b-deficient mice showed enhanced sLTD induced by a low-frequency stimulation (LFS; 2Hz/10min), compared to littermate controls (Figure 3A). Taken together, our LTP and LTD data in one month old animals suggest that SNAP-25a favors the expression of sLTD over sLTP in the developing brain, consistent with a previous study [29] in which we showed that LTP at Schaffer collateral-CA1 synapses is reduced in both male and female SNAP-25b-deficient mice at one month of age. In contrast, four month old SNAP-25b deficient mice displayed sLTD that did not differ from their littermate controls (Figure 3B). These results also suggest that compensatory effects can restore both LTP and LTD to control levels and balance in adult mice lacking SNAP-25b.

### 2.3. Place Avoidance Spatial Learning

A recent study from our group found, in an active avoidance spatial learning test, that the same one month old SNAP-25b-deficient mice showed impaired learning acquisition rates, and slower extinction of learning once acquired, suggesting weaker initial learning and greater flexibility for relearning new contingencies [29]. Our current findings suggest that compensatory mechanisms have returned LTD and LTP to near control levels by four months of age in mice lacking the adult SNAP-25b isoform, leading us to test whether behavioral phenotypes found in younger mice lacking SNAP-25b are also compensated in adulthood.

**Figure 4:**
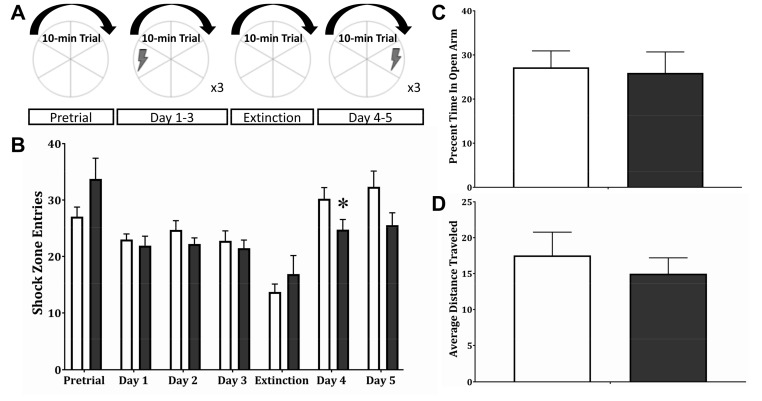
Active avoidance initially shows altered cognitive flexibility in adult SNAP-25b-deficient mice of both sexes with no difference in anxiety-like behaviors or locomotion. (**A**): Active avoidance training schedule. Pretrial and Extinction: 1–10 min trial without shock. Initial learning: 3 days of 3–10 min trails/day with a left shock zone. Conflict learning: 2 days of 3–10 min trials/day with a 180° shifted shock region. (**B**): Active avoidance assay average daily counts of shock zone entries. Four month old SNAP-25b-deficient mice (black bars, *n* = 12) learned to avoid the original shock zone as well as controls (white bars, *n* = 17). After extinction, four month old SNAP-25b-deficient mice initially learned faster and entered the new shock zone fewer times than WT littermates (day 4; *p* < 0.05; one-way ANOVA for repeated measures). (**C**): The same four month old mice deficient in SNAP-25b (black bar, *n* = 12) showed no difference in time spent in the open arm of the elevated plus maze (EPM) relative to controls (white bar, *n* = 17). Therefore, no difference in anxiety-like behavior was detected. (**D**): These older SNAP-25b-deficient mice (black bar, *n* = 12), compared to WT littermates (white bar, *n* = 17), also showed no difference in general locomotion, indicated by total distance traveled during EPM assessment. Each point is mean + SEM of n animals.

To evaluate the behavioral phenotypes associated with a lack of SNAP-25b throughout developmental into adulthood, we used an active place avoidance assay developed by Fenton and colleagues [31]. In this task, rodents are placed on a turning metal grid platform and, using spatial cues, must learn to move to avoid a shock given when the animal enters one segment of the circular field (Figure 4A). Littermate controls and SNAP-25b-deficient mice were subject to multiday habituation, training trials, extinction and conflict discrimination as described [31]. In contrast to deficits in this learning, as previously observed in one month old mutant mice, adult mice lacking SNAP-25b showed no differences in the initial learning phase in days 1–3 (Figure 4B). However, after extinction (day 4), when the shock zone was shifted 180° from its initial position, SNAP-25b-deficient mice entered the new shock zone fewer times than controls, suggesting a more rapid relearning of the new shock zone location. This may reflect a behavioral learning flexibility. No difference in anxiety-like behavior was detected in four month old SNAP-25b deficient mice (Figure 4C), using an elevated plus maze. Increased anxiety observed at one month in our previous study was no longer detected in older SNAP-25b-deficient mice. Finally, motor function as assessed by total path length was not different in SNAP-25b-deficient mice and WT littermates (Figure 4D). These data indicate that adult SNAP-25b-deficient mice have compensated for the impairments in synaptic plasticity and learning at one month of age, consistent with the shift from a reduction in LTP at one month of age, to larger LTP at four months of age, and the ‘renormalization’ of the magnitude of LTD reached by adulthood.

### 2.4. Effects of Learning Acquisition on Long-Term Synaptic Plasticity

**Figure 5:**
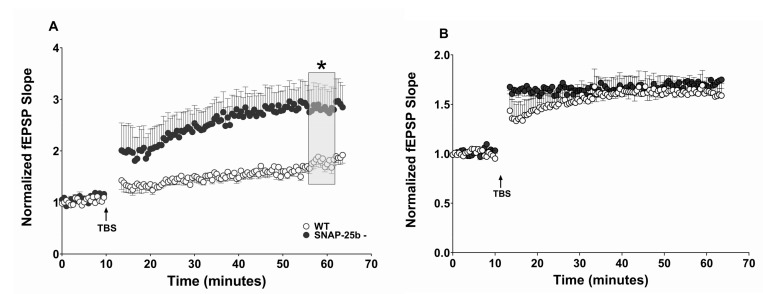
Hippocampal dependent training alters LTP in adolescent SNAP-25b-deficient mice of both sexes. (**A**): Time course of TBS (arrows) used to induce LTP in younger mice. After completing an active avoidance assay, adolescent mice lacking SNAP-25b showed enhanced LTP (filled circles, *n* = 7) relative to controls (open circles, *n* = 11). (* *p* < 0.01; Student’s t-test). Each point is normalized to the averaged baseline and is mean ± SEM of n slices; (**B**): TBS LTP (arrows) recorded from area CA1 in four month old mice that had completed a multiday active avoidance training. SNAP-25b-deficient mice (filled circles, *n* = 18) expressed LTP at levels similar to controls (open circles, *n* = 18). However, SNAP-25b deficient mice did show significantly larger PTP. Each point is normalized to the averaged baseline and is mean ± SEM of n slices.

In a cohort of mice that went through active place avoidance behavioral testing, sLTP and sLTD were assessed post-training, to determine the impact of hippocampal-dependent training of place avoidance on hippocampal synaptic transmission and long-term activity-dependent plasticity in SNAP-25b-deficient mice. Once mice completed all five days of the training paradigm, they were sacrificed and used for slice electrophysiology recordings. In contrast to untrained mice, one month old SNAP-25b-deficient mice exhibited enhanced sLTP compared to littermate controls (Figure 5A). Prior to training, the same SNAP-25b-deficient mouse line had displayed reduced LTP at Schaffer collateral-CA1 synapses. While the response to HFS post-training was larger in SNAP-25b-deficient mice at one month of age, the magnitude of sLTP was not altered by behavioral training in four month old SNAP-25b deficient mice (Figure 5B).

**Figure 6:**
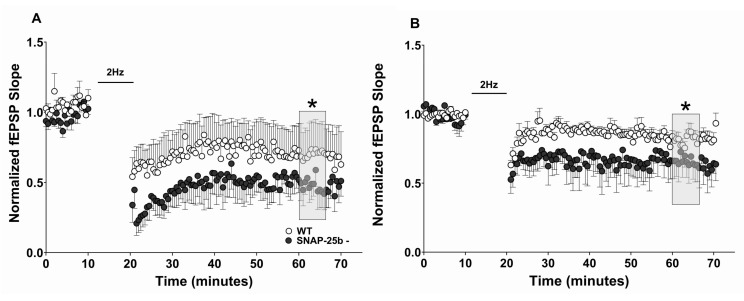
LTD is enhanced in young and old SNAP-25b-deficient mice of both sexes by hippocampal-dependent training. (**A**) One month old SNAP-25b-deficient mice (filled circles, *n* = 7) exhibited significantly larger sLTD compared to WT littermates (open circles, *n* = 4). (*, *p* < 0.01; Student’s *t*-test). Bar indicates 2Hz/10mins LTD stimulus application. Each point is normalized to the averaged baseline and is mean ± SEM of n slices; (**B**) Four month old SNAP-25b-deficient mice (filled circles, *n* = 6) also exhibited greater sLTD than WT controls (open circles, *n* = 13, *p* < 0.05; Student’s t-test). Bar indicates 2 Hz/10 min LTD LFS stimulus train. Each point is normalized to the averaged baseline and is mean ± SEM of n slices.

Unlike adolescent mice lacking SNAP-25b, sLTD was altered in four month old adult SNAP-25b-deficient mice compared to WT littermate controls only after the passive avoidance spatial learning task (Figure 6B). Prior to training, four month old SNAP-25b-deficient mice did not show altered sLTD, while younger mice showed the same enhancement in sLTD relative to controls both before and after passive avoidance training. These data show that, in one month old mice lacking SNAP-25b, reductions in LTP and increases in LTD are both present, while by four months of age, compensatory mechanisms have returned LTD levels to normal. Nevertheless, the effect of learning acquisition on adult mice expressing only the juvenile SNAP-25a isoform was to upregulate both LTP and LTD at Schaffer collateral-CA1 synapses.

### 2.5. mGluRII and NMDA Receptor-Dependent LTD of Synaptic Transmission

We investigated if changes in activation of glutamate receptors might be responsible for differences observed in synaptic plasticity resulting from a lack of SNAP-25b. Multiple reports indicate an essential role for SNAP-25 in transient presynaptic suppression of transmitter release by the 5-HT serotonin receptor at lamprey synapses [32], and in mGluRII-dependent chemical- and stimulus-evoked LTD at Schaffer collateral-CA1 hippocampal synapses. Evidence points to an interaction between the C-terminus of SNAP-25 with the G-protein Gβγ being necessary for suppression of neurotransmitter release at all of these synapses. C-terminus cleavage of SNAP-25 by botulinum toxin A prevents Gβγ and SNAP-25 interaction necessary for depression of transmitter release [32,33], as does infusion of a C-terminal fragment of SNAP-25 that binds to Gβγ [34]. Given the role of these glutamate receptors in the induction of LTD of presynaptic transmitter release, we tested whether there were differences in the expression of mGluRII or NMDAR chemical LTD (cLTD) produced by a lack of SNAP-25b. To evaluate the involvement of each form of presynaptic cLTD and how they interface with SNAP-25 to affect presynaptic vesicular release, mGluRII- and NMDAR-dependent LTD were selectively induced in hippocampal slices from one and four month old mice lacking SNAP-25b, by bath application of either NMDA (20µM) or the mGluRII agonist DCG-IV (25 µM).

**Figure 7:**
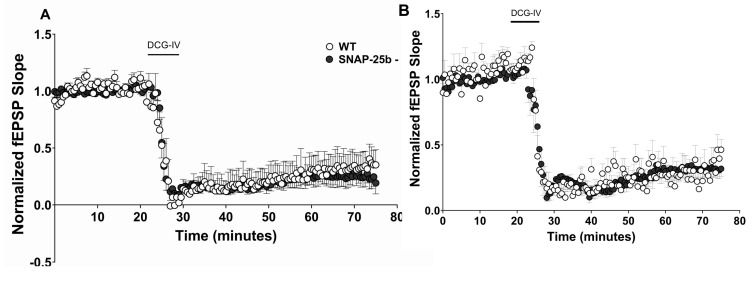
One and four month old SNAP-25b-deficient mice of both sexes show no differences in mGluRII-dependent cLTD. Time course of mGluRII cLTD induced by the receptor agonist DCG-IV (25µM; solid bar = 5 mins at 2mL/min) at Schaffer collateral-CA1 synapses. Each point is normalized to the averaged baseline and is mean ± SEM of n slices. (**A**): In one month old SNAP-25b-deficient hippocampal slices (filled circles, n=11), mGluRII LTD was not altered compared to littermate controls (open circles, *n* = 7); (**B**): In four month old SNAP-25b-deficient (filled circles, *n* = 7) and WT (open circles, *n* = 9) mice, mGluRII LTD did not differ in magnitude.

In mGluRII cLTD, lack of SNAP-25b did not alter the amplitude of mGluRII cLTD in either one (Figure 7A) or four month old (Figure 7B) SNAP-25b-deficient mice compared to WT littermate controls, indicating that molecular mechanisms downstream of synaptic stimulation underlie the induction of mGluRII-dependent presynaptic LTD. Moreover, they are not differentially regulated by the two isoforms of SNAP-25.

**Figure 8:**
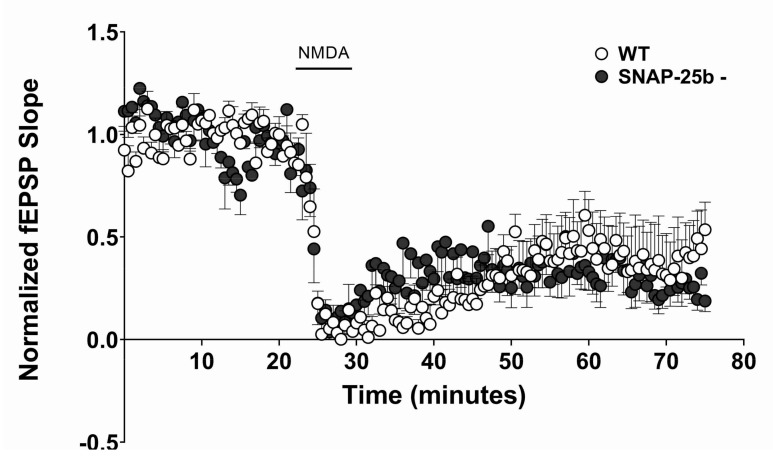
Differences observed in synaptic plasticity in one month old SNAP-25b-deficient mice of both sexes were not due to alterations in NMDAR response to glutamate. The presence of SNAP-25a in mutant mice (filled circles, *n* = 5) did not alter the extracellular amplitude of NMDA-induced cLTD (20 µM; solid bar = 5 mins at 2mL/min) compared to WT littermates (open circles, *n* = 7) at Schaffer collateral-CA1 synapses. Each point is normalized to averaged baseline and is mean ± SEM of n slices.

NMDAR cLTD also exhibited the same amplitude of fEPSP in one month old SNAP-25b-deficient mice as in control mice (Figure 8). If components of the NMDAR cascade interact with SNAP-25, this interaction does not appear to be influenced by a lack of SNAP-25b in adolescent mice. Further, we found that NMDAR-dependent LTD could not be elicited in either mutant or control mice by bath application of NMDA at four months of age, indicating that synaptic stimulation is an essential component for the induction of NMDAR-dependent LTD in adult mice, since the majority of stimulus-evoked LTD is blocked by NMDAR antagonists. Taken together, these findings suggest that the ‘a’ and ‘b’ isoforms of SNAP-25 differentially regulate the induction of LTD through regulation of the magnitude and patterns of synaptic stimulation and through its effects on presynaptic terminals that require synaptic activity, rather than by altering the downstream activation of glutamate receptors, either post- or presynaptically.

## 3. Discussion

SNAP-25 is an essential SNARE protein and a regulatory target for the expression of short- and long-term plasticity of presynaptic transmitter release. The individual contributions of each isoform differ in regulation of synaptic transmission and activity-dependent long-term synaptic plasticity [29]. Given the importance of LTP and LTD in activity-dependent networks that underlie learning and memory [35,36], we assessed alterations in LTP, LTD and behavioral learning in SNAP-25b-deficient mice, compared to WT mice that exhibited normal developmental regulation of the ‘a’ and ‘b’ isoform. Although relative expression levels of SNAP-25 isoforms throughout different brain regions are not yet fully evaluated, the switch from SNAP-25a to SNAP-25b is well described in the hippocampus. We used Schaffer collateral-CA1 synapses in hippocampal slices to assess LTP and LTD, and a hippocampus-dependent behavioral task, to correlate the observed changes with expression of SNAP-25a or SNAP-25b as the dominant isoforms at that time in development.

The reduced level of LTP and enhanced magnitude of LTD expressed in one month old SNAP-25b-deficient mice relative to littermate controls suggests that SNAP-25a favors the induction of LTD over LTP, and is consistent with our previous study showing that both male and female SNAP-25b-deficient mice exhibited significantly smaller LTP [29]. By preventing a down regulation of LTD during development, the absence of SNAP-25b could prevent the shift in synaptic plasticity favoring larger LTP that normally occurs around three weeks of age. During the first four weeks of development, WT mice display a dramatic increase in SNAP-25b mRNA expression, and this corresponds to a developmental window for complete maturation of synaptic connections. Therefore, the loss of SNAP-25b could alter synaptic maturation by modifying activity-dependent synaptic plasticity. In this study, SNAP-25b-deficient mice showed a delayed progression in normal expression levels of LTP and LTD. It is probable that, without the dramatic increase in SNAP-25b, which markedly decreases the ratio of SNAP-25a to SNAP-25b, one month old SNAP-25b-deficient mice were more like younger WT mice. In the latter mice the ratio of SNAP-25a/SNAP25b is still high, and LTD is favored over LTP. This observation is in line with previous reports that SNAP-25a is less efficient at coordinating bursts of vesicular release [30]. The results of our study support the conclusion that the switch from SNAP-25a to SNAP-25b may be a key mediator of the developmental switch in relative magnitudes of LTD and LTP seen in the hippocampus during development.

By four months of age, SNAP-25b-deficient mice appeared to have over-compensated for the lack of SNAP-25b. In the process of up-regulating the strength of LTP to match adult WT LTP levels, hippocampal Schaffer collateral-CA1 synapses in SNAP-25b-deficient brain slices exhibited enhanced LTP relative to WT controls. It is intriguing that these synapses also showed larger PTP immediately after HFS. The increase in PTP may be due to increased residual calcium in the presynaptic terminal after HFS, suggesting the possibility of alterations in presynaptic calcium regulation that increase vesicular release immediately after HFS. An enhancement of both PTP and LTP could also signify an upregulation of NMDAR-gated postsynaptic currents. In particular, NR2B subunit containing NMDARs can be upregulated, resulting in enhanced LTP [37,38].

In contrast, levels of LTD in these older SNAP-25b-deficient mice were similar to controls, suggesting that, if NR2B NMDAR subtypes are lesser participants in the induction of LTD, presynaptic sites of regulation may account for compensatory shifts in expression of LTD in older mice. With regard to the amplitude of LTD, SNAP-25b-deficient mice were able to accurately compensate for the enhancement seen in younger mice, bringing LTD back to levels observed in littermate controls. Taken together, findings at one and four months of age confirm the hypothesis that the developmental shift from SNAP-25a to SNAP-25b is an important factor in regulating the relative magnitude of sLTP and sLTD in the developing and adult brain. Findings from previous studies and the present study (highlighted in grey) of these SNAP-25b mice at 1–4 months of age are summarized in Table 1.

It is unclear whether compensatory mechanisms underlying shifts in LTP and LTD result from a lack of SNAP-25b or over-expression of SNAP-25a. Although the adolescent ‘a’ isoform is less effective at coordinating vesicle fusion, it can rescue SNAP-25 null neurons in culture and coordinate synchronous stimulus evoked release [39]. The absence of SNAP-25b may promote enhanced functionality or increased recruitment of SNAP-25a or altered association of SNAP-25a with accessory proteins as mice age. Munc18-1, an accessory SNARE protein which promotes SNARE complex formation, also interacts with SNAP-25 and binds to SNAP-25b more easily than SNAP-25a [23]. Differential interaction of SNAP-25 isoforms with Munc18-1 could potentially alter the rate of SNAP-25a incorporation into SNARE complexes during vesicle priming. As mice age and more SNAP-25a is incorporated into SNARE complexes, more vesicles may be accessible for synaptic transmission and account for the compensatory effects seen in older SNAP-25b-deficient mice. To test this possibility, it would be helpful to investigate changes in the function and size of the readily-releasable vesicle pool (RRP) in SNAP-25b-deficient mice at one and four months, using imaging of FM1-43 release and electron microscopy. Alternatively, these compensatory mechanisms may be postsynaptic in origin, involving changes such as up-regulation of NR2B-NMDAR expression to compensate for continued impairments in presynaptic function.

At the same time, SNARE proteins can also play significant roles in postsynaptic vesicle-mediated trafficking, including trafficking of NMDAR [40]. Hussain and colleagues have recently shown that SNAP-25 is also present in the postsynaptic density, although its location was not altered one hour after induction of LTP [41]. At this time, an additional postsynaptic role for SNAP-25 in developmental regulation of long-term synaptic plasticity cannot be ruled out.

To assess whether hippocampal-dependent learning and memory is affected by lack of the adult SNAP-25b isoform throughout development, hippocampal-dependent spatial learning was evaluated in four month old SNAP-25b-deficient mice using an active avoidance assay. Four month old SNAP-25b-deficient mice initially showed no differences in acquisition of learned place avoidance, but they did show enhanced conflict learning immediately after extinction, i.e., a more rapid shift away from a previous shock zone to learn a new zone. On the second day of conflict reversal learning, there was only a nonsignificant trend towards fewer shock zone entries for the mice lacking SNAP-25b. The possibility exists that, since older SNAP-25b-deficient mice did not experience the initial delay in learning we observed in younger mice, their underlying memory deficit had largely disappeared as compensatory mechanisms were expressed during development. Given that, at one month of age, this same mouse line showed impaired cognitive learning, we hypothesize that enhanced LTD in these adolescent animals accounted for their performance during the relearning phase of the test, and both LTD and learning had returned to WT levels in adulthood. The hypothesis of compensatory changes in older SNAP-25b-deficient mice is also supported by our earlier study, in which SNAP-25b-deficient mice exhibited higher escape latencies in the hippocampal-dependent Morris water maze behavioral task, although anxiety levels were significantly higher in the elevated plus maze test [29].

To address any underlying memory deficit not detected by the active avoidance assay, additional behavioral learning and memory paradigms will be needed to test hippocampus-dependent and -independent forms of retention and relearning, to determine if behavioral flexibility is altered by the subtle differences in presynaptic function afforded by the isoforms of SNAP-25. For example, it is possible to increase the complexity of the spatial avoidance learning task by adding a rotating shock segment with the stationary segment during the initial learning phase. Mice expressing only SNAP-25a exhibited a higher rate of neurogenesis, as shown by a higher number of doublecortin-positive precursor cells [14]. Increased neurogenesis may lead to improved performance on cognitive flexibility tasks and could serve as an alternative explanation for the results observed here.

Prior to training in the active avoidance task, mice were monitored for anxiety. The time spent in the open arm of the elevated plus maze relative to total time in the apparatus was used as an indicator of anxiety. At four months of age, SNAP-25b-deficient animals were no different from controls and showed comparable levels of locomotion, indicating that our spatial learning findings cannot be attributed to alterations in motivation or motor function.

Some of the same mice that went through active avoidance behavioral testing, referred to as trained mice, were then monitored for changes in sLTP and sLTD. Trained one month old SNAP-25b-deficient mice expressed enhanced, instead of reduced, LTP, but still showed the larger LTD relative to littermate controls that we observed in untrained mice. The relative switch in the strength of LTP in trained versus untrained mice lacking SNAP-25b suggests that the act of training functionally improved synaptic plasticity, or that SNAP-25b-deficient mice demonstrate less LTP elicited by active avoidance training itself, leaving more room below ceiling levels for LTP to be elicited by HFS.

In contrast to adolescent mice, adult trained mice exhibited LTP similar in magnitude to controls, but LTD was still significantly enhanced in magnitude. In untrained SNAP-25b-deficient mice at one month of age, LTP was lower than controls. However, by four months of age, untrained SNAP-25b-deficient mice showed enhanced LTP. Since adolescent, untrained SNAP-25b-deficient mice exhibited smaller LTP than controls, compensatory mechanisms may have been activated. This may have resulted in overshooting the normal homeostatic set point in naive mice, leading to larger LTP in untrained SNAP-25b-deficient mice. Conceivably, the training paradigm could have activated cascades that reset synapses to normal levels of LTP, and a secondary consequence of this compensation to adjust LTP may have caused an enhancement of LTD. Moreover, the difference seen in SNAP-25b-deficient mice post training could have been due to a shift in the ability to regulate the sliding threshold of the synapse. If the sliding threshold scale is being altered in SNAP-25b-deficient mice as a compensatory mechanism to accommodate the deficits in synaptic plasticity, it could imply that SNAP-25 function contributes to a synapse’s ability to regulate metaplasticity or the threshold for changing synaptic strength.

To evaluate the effects of lack of SNAP-25b on baseline transmitter release, we measured presynaptic neurotransmitter release with FM1-43. The destaining kinetics of this vesicle-specific dye indicated a faster rate of neurotransmitter release. This result can either be interpreted as i) higher baseline release probability in mice lacking SNAP-25b or ii) a smaller pool of RRP, as described by [30].

Impaired transmitter release mechanisms may result in memories that are not as persistent and stable as in WT mice, making them more susceptible to reversal in conflict learning assays. Different transmitter release probabilities are suggested to impair the induction of LTP, resulting in weaker synaptic strengthening and memory formation. Therefore, extinction in adult SNAP-25b-deficient mice may proceed faster than in control mice that express adult SNAP-25b.

We have previously shown that mutations in the C-terminus of SNAP-25 in a gene targeted mouse model enhances the magnitude of LTP at Schaffer collateral-CA1 synapses [42]. This is because of the reduced interaction between SNAP-25 and the inhibitory G_i/o_ proteins [43]. The absence of SNAP-25b did not alter the amplitude of mGluRII-dependent LTD measured at Schaffer collateral-CA1 synapses when DCG-IV was bath applied, or NMDAR-dependent LTD when NMDA was bath applied to hippocampal slices from such mice compared to four month old littermates. This suggests that SNAP-25a and Gβγ can interact to induce cLTD in adult SNAP-25b-deficient mice with similar affinity as in WT mice during both adolescence and adulthood. These results are not consistent with recent immunoprecipitation data showing that SNAP-25a-containing SNARE complexes associate with Gβγ 50% less than SNAP-25b-containing complexes [23]. Since a difference in chemically induced NMDA or mGLuRII-dependent LTD was not detected, even if Gβγ is less likely to interact with SNAP-25a, it appears that lower levels of Gβγ and SNAP-25 interaction are still sufficient to permit expression of normal magnitude of NMDAR and mGLuRII cLTD that is downstream of, and does not depend upon, glutamate release. These results also imply that the altered synaptic plasticity induced by stimulus in young and old transgenic mice versus WT mice may have been in response to altered presynaptic activity, since we observed no difference in fEPSPs when mGluRII-LTD was expressed in SNAP-25b-deficient mice. Our previous work in rats has shown that presynaptic infusion of Gβγ scavenging peptides, ct-SNAP-25 and structurally distinct mSIRK, can each occlude mGluRII presynaptic LTD [44]. Therefore, Gβγ binding to SNAP-25a in mice expressing only this isoform may show larger sLTD compared to littermate controls.

In summary, one role of the switch from expression of the SNAP-25a isoform to SNAP-25b isoform seems to be to down-regulate the predominance of LTD of synaptic strength in early activity-dependent long-term plasticity, favoring an up-regulation of LTP that promotes faster learning acquisition and retention, but reducing behavioral flexibility when learning contingencies change.

## 4. Materials and Methods

### 4.1. Animals

SNAP-25b deficient on C57BL/6NCrl background and age-matched wild type littermate mice of both sexes were generated as described previously [14] in the laboratory of Dr. Christina Bark at the Karolinska Institute, Stockholm, Sweden, and genotyped at NYMC by PCR using published methods [14]. A colony of these mice was bred and maintained in the AALAC-accredited animal facility of New York Medical College in accordance with AALAC standards and applicable guidelines. All breeding, genotyping, electrophysiological and behavioral experiments were performed at New York Medical College according to AAALAC International standards and guidelines and approved by the Institutional Animal Care and Use Committee of New York Medical College, Valhalla, NY, USA (IACUC Ethical Permit # 25-12-0312, approved March 20, 2013 and # 91-2-1012, approved October 16, 2012).

### 4.2. Hippocampal Slice Preparation and Recording

Coronal brain slices were used to analyze dorsal hippocampal Schaffer collateral-CA1 synapses. Brains were removed from male and female mice decapitated under deep isoflurane anesthesia, and immersed in ice-cold (2–4 °C) oxygenated sucrose-containing artificial cerebrospinal fluid (ACSF) in mM: NaCl 87, NaHCO_3_ 25, NaH_2_PO_4_ 1.25, KCl 2.5, CaCl_2_ 0.5, MgCl_2_ 7, d-glucose 25 and sucrose 75, (saturated with 95% O_2_/5% CO_2_, pH 7.4). The cerebellum and frontal lobes were removed, brains were hemisected, and each half was mounted on a metal stage with cyanoacrylate glue and again submerged in ice-cold sucrose-containing ACSF. Coronal slices 350–400 μm thick were cut with a vibratome (VT1200S, Leica Microsystems, Buffalo Grove, IL, USA), placed in an interface holding chamber at 32 ± 2 °C for at least 30 min, and then transferred to room-temperature ACSF in mM: NaCl 126, NaHCO_3_ 26, NaH_2_PO_4_ 1.25, KCl 3, CaCl_2_ 2.5, MgCl_2_ 2 and d-glucose 10 (constantly bubbled with 95% O_2_/5% CO_2_, pH 7.4) for an additional 30 min before start of recording. Slices were maintained at room temperature until being moved to an interface recording chamber and maintained thereafter at 32 ± 2 °C for the remainder of the experiment.

Slices were continuously perfused at a rate of 2 mL/min with oxygenated normal ACSF. Field excitatory postsynaptic potentials (fEPSPs) were evoked in the CA1 area by activation of Schaffer collateral axons using a stainless steel bipolar stimulating electrode placed in the stratum radiatum. A thin-walled glass microelectrode was pulled (1–2 MΩ; Flaming/Brown micropipette puller, Model P-97, Sutter Instruments, Novato, CA, USA) and filled with normal ACSF. The borosilicate micropipette was placed approximately 200–300 µm from the stimulating electrode in the similar stratum radiatum area touching distal dendritic arbors to record fEPSPs. A constant current stimulation was applied by an ISO-Flex isolator driven by a Master-8 programmable pulse generator (A.M.P.I, Jerusalem, Israel) and recorded at half maximal fEPSP amplitude once every 30 s. Recordings were amplified by Multiclamp 700B Axon Instruments (Molecular Devices, San Jose, CA, USA), digitized by an AD board (National Semiconductor, Santa Clara, CA, USA), and analyzed using SciWorks software (DataWave Technologies, Loveland, CO, USA) by calculating the maximum slope within 20–80% of the maximum fEPSP initial negative slope. At least 15-min stable baselines were recorded at 0.033 Hz prior to application of either high-frequency theta burst stimulation (TBS; 4 trains of 10 bursts of 5 pulses each at 100 Hz with a 200 msec interburst interval, applied at 3 min intervals) to induce LTP, or a single train of low-frequency stimuli (LFS; 2Hz/10min) to induce LTD.

### 4.3. Two-Photon Laser Scanning Microscopy of FM1-43 Vesicular Release from Schaffer Collateral Presynaptic Terminals

Fluorescence was visualized using a customized two-photon laser-scanning Olympus BX61WI microscope with a 60x/0.90W water immersion infrared objective lens and an Olympus multispectral confocal laser scan unit. The light source was a Mai-Tai™ laser (Solid-State Laser Co., Mountain View, CA, USA), tuned to 820 nm for exciting FM1-43. Epifluorescence was detected with photomultiplier tubes of the confocal laser scan head with pinhole maximally opened and emission spectral window optimized for signal over background. In the transfluorescent pathway, a 565 nm dichroic mirror was used to separate green and red fluorescence to eliminate transmitted or reflected excitation light (Chroma Technology, Rockingham, VT, USA). After confirming the presence of Schaffer collateral-evoked fEPSPs >1 mV in amplitude in CA1 stratum radiatum, 10 µM 6-cyano-7-nitroquinoxaline-2,3-dione (CNQX) was bath-applied throughout the rest of the experiment to prevent synaptically driven action potentials in the pyramidal neurons and prevent the accelerated dye release. Presynaptic boutons were loaded by bath-applying 5 µM FM1-43 (Invitrogen Molecular Probes, Eugene, OR, USA) in hypertonic ACSF supplemented with sucrose to 800 mOsm for 25 s to selectively load the readily releasable pool (RRP), then returned to normal ACSF. Stimulus-induced destaining was measured after 30 min perfusion with dye-free ACSF, by 0.1 Hz bipolar stimuli (150 µs DC pulses).

The technique of loading presynaptic vesicles with a lipophilic styryl dye permits analysis of vesicle fusion dynamics in presynaptic terminals [45], since FM1-43 preferentially loads into presynaptic vesicles. Once the dye is loaded into the vesicles, it can only be released when vesicles fuse with the membrane and discharge their contents. Two-photon analysis of FM1-43 makes it possible to directly image presynaptic rates of vesicular release [46].

### 4.4. Elevated Plus Maze

The elevated plus maze (EPM) is used as a general indicator of anxiety. The apparatus consists of two open arms (50 × 10 cm) and two closed arms (50 × 10 × 40 cm), connected by a center platform (10 × 10 cm) made of opaque dark grey plexiglass (Stoelting Co., Wood Dale, IL, USA). The arms of the EPM are elevated 50 cm above the floor. Animals (*n* = 12–17) were placed in the center platform of the EPM, facing an open arm and allowed to explore the maze for 5 min. The middle point of the animal was used as the reference point to determine the position of a mouse and recorded using AnyMaze behavioral analysis software (Stoelting Co., Wood Dale, IL, USA). Percent of time spent in the open arms was calculated, where a decrease in percent of time spentg in the open arms indicates an anxiety phenotype. The software also measured total distance travelled during EPM assessment.

### 4.5. Active Place Avoidance Learning Task

The active place avoidance task used was previously described by Burghardt et al. [31]. In this paradigm, mice are placed on a circular rotating platform that continuously turns clockwise at a speed of 1 rpm. Over several days and multiple trials (Figure 3a), mice (*n* = 12–17) learn to identify the 60° shock zone guided by spatial markers on the walls surrounding the apparatus. Entrance into the shock zone triggered a brief foot-shock (500 ms, 60 Hz, 0.2 mA) with an inter-shock interval of 1.5s that would cease upon leaving the shock zone. The middle point of the animal was used as the reference point to determine the position of a mouse and recorded using AnyMaze behavioral analysis software (Stoelting Co., Wood Dale, IL, USA) Using the same software, the number of shock-zone entries was measured, where a decrease in shock-zone entries indicates learning. During the initial pre-training trial (10 min) when the shock was turned off, mice were allowed to habituate to the apparatus and showed no preference for any area of the platform. Subsequently, the shock was turned on, and the mice completed three training sessions (10 min each) per day for three days, followed by an extinction trial (10 min) on the next day when the shock was turned off and the animals were allowed to ambulate freely into the zone previously associated with the shock. After extinction, a conflict variant task was performed in order to test cognitive flexibility. The shock zone was moved 180° from where the original shock zone was placed and three conflict-training sessions (10 min each) were conducted per day for two days. Mice had to avoid the new shock zone which requires cognitive flexibility and is represented in this task by the simultaneous suppression of the learned condition response of avoiding the original shock zone and learning the new association of a foot-shock with a different zone.

### 4.6. Statistical Analysis

In LTP and LTD experiments, comparison of fEPSP slopes normalized to baseline slopes from slices from wildtype and SNAP-25b-deficient mice were analyzed by two-tailed Student’s *t*-test for unpaired data. The null hypothesis was that true mean normalized slope between wildtype and SNAP-25b-deficient slices was equal to zero, the alternative hypothesis was that the true mean difference was not equal to zero. One-way ANOVA with repeated measures was used to determine differences between WT and SNAP-25b-deficient mice for all behavioral testing, followed by Bonferroni correction for multiple comparisons (Prism, GraphPad Software, San Diego, CA, USA). All data points are mean ± SEM of n slices, recording from 1–2 slices per mouse. Significance level was pre-set to *p* < 0.05.

## 5. Conclusions

The isoform SNAP-25a, which is expressed first developmentally in rodents, developmentally regulates the expression of both long-term depression (LTD) and long-term potentiation (LTP) of synaptic strength at Schaffer collateral-CA1 synapses in the hippocampus. In one month old mice lacking the adolescent/adult isoform SNAP-25b, Schaffer collateral-CA1 synapses showed faster release kinetics, decreased stimulus-evoked LTP and enhanced LTD, while chemically induced mGluR-dependent and NMDAR-dependent LTD not requiring synaptic stimulation were not affected. By four months of age, SNAP-25b-deficient mice had compensated for the lack of adult SNAP-25b, exhibiting larger LTP and normal LTD compared to wild type littermates. These compensatory changes restored normal learning and memory in adult mice, as well as enhancing reversal learning. These data indicate a connection between the regulation of long-term synaptic plasticity and development of cognitive learning capacity. The recovery of plasticity and learning in adulthood indicates mechanisms that can compensate for a lack of the adult SNAP-25b isoform.

## Figures and Tables

**Table 1:** Developmental Effects of SNAP-25b Deficiency.

Developmental Effects of SNAP-25b Deficiency					
Postnatal age	LTP	LTD	Basal synaptic transmission (I/O, PPF, Pr)	Active Avoidance Learning	Metabolic and Expression Effects
1 month old	Reduced LTP (1,2)	Larger LTD (2) No diff in mGluRII-dependent LTD (2) No diff in NMDAR-dependent LTD (2)	Higher ceiling fEPSP baseline amplitude (1) Reduced PPF and increased Pr in males, but not females (1) Faster presynaptic vesicular release (2)	Impaired (1) Enhanced LTP, but did not alter LTD (remained larger than controls)	WT females higher SNAP-25a expression levels than WT males, suggesting SNAP-25b switch may occur later in females
2 month old	Reduced LTP (1)		Reduced PPF and increased Pr in males, but not females (1)		Decreased interaction of SNAP-25 with MUNC-18 and Gβ_2_ subnits (3) Increased insulin secretion from β–islet cells (4) Slowed/prolonged glucose-stimulated Ca^2+^ increases and desynchronized oscillations in β–islet cells (4) Metabolic disease (obesity, hyperglycemia, liver steatosis, adipocyte hypertrophy) and enhanced response to a high-fat diet (5)
4 month old	Increased LTP (2)	No diff in LTD (2) No diff in mGluRII-dependent LTD (2)		Rescued LTP to control levels and LTD larger than controls (2)	

CITATIONS: (1) Irfan et al., Scientific Reports, 9:6403,2019; (2) Gopaul et al., this study; (3) Daraio et al., Neurosci Lett, 674: 75–80; (4) Daraio et al., Scientific Reports, 7:7744, 2017; (5) Valladolid-Acebes et al., PNAS, 112: E4326–4335. Abbreviations: LTP (long-trem potentiation), LTD (long-term depression), mGluR1 (metabotropic glutamate receptor 1), NMDAR (N-methyl-D-aspartate receptor), PPF (paired-pulse facilitation), I/O (input/output relations), Pr (release probability), WT (wild type).

## Abbreviations

EPM	Elevated plus maze
LTD	Long-term depression
LTP	Long-term potentiation
mGluR	Metabotropic glutamate receptor
NMDAR	N-methyl-d-aspartate glutamate receptor
RRP	Readily releasable vesicle pool
SNAP-25	Synaptosomal-associated protein of 25 kD
SNARE	Soluble N-ethylmaleimide–sensitive factor Attachment protein REceptor
VAMP2	Vesicle-associated membrane protein 2
